# Maternal participation in a nutrition education program in Uganda is associated with improved infant and young child feeding practices and feeding knowledge: a post-program comparison study

**DOI:** 10.1186/s40795-017-0140-8

**Published:** 2017-04-04

**Authors:** S. B. Ickes, C. Baguma, C. A. Brahe, J. A. Myhre, L. S. Adair, M. E. Bentley, A. S. Ammerman

**Affiliations:** 1 The University of Washington Department of Health Service and Nutritional Sciences, University of Washington School of Public Health, Raitt Hall, Box 353410, Seattle, WA 98195-3410 USA; 2 Mbarara University of Science and Technology, Mbarara, Uganda; 3 Georgetown Medical School, Georgetown, University, Washington, DC USA; 4 Serge and Naivasha Health Center, Naivasha, Kenya; 5 Department of Nutrition, Gillings School of Global Public Health, The University of North Carolina at Chapel Hill, Chapel Hill, NC USA

**Keywords:** Feeding Practice, Nutrition Education, Complementary Food, Nutrition Education Program, Animal Source Food

## Abstract

**Background:**

Cost-effective approaches to improve feeding practices and to reduce undernutrition are needed in low-income countries. Strategies such as nutritional counseling, food supplements, and cash transfers can substantially reduce undernutrition among food-insecure populations. Lipid-based nutrient supplements (LNS) are an increasingly popular strategy for treating and preventing undernutrition and are often delivered with nutrition education. The post-program effects of participation in a LNS-supported supplemental feeding program on Infant and Young Child Feeding (IYCF) practices and caregiver child feeding knowledge are not well understood. The objective of this study was to understand whether children’s diet quality and caregiver nutrition knowledge was improved after participation in such a program.

**Methods:**

We conducted a post-program comparison group study to compare feeding practices and caregiver nutrition knowledge among mother-child dyads who completed a nutrition education program and a community comparison group in western Uganda. We administered a feeding practices survey and two 24-h dietary recalls to 61 Post-Program (PP) caregivers and children ages 6 to 59 months (mean age = 25.1 months) who participated in a supplemental feeding program (which included growth monitoring, caregiver nutrition education, and LNS) and a Comparison Group (CG) of 61 children and caregivers. PP caregivers were recruited 4 to 8 weeks after program participation ended. We hypothesized that PP caregivers would report better IYCF practices and greater knowledge of key nutrition education messages related to IYCF.

**Results:**

PP children had higher dietary diversity scores (3.0 vs 2.1, *p =*0.001) than CG children, and were more fed more frequently (3.0 vs 2.1 times per day, *p* = 0.001). IYCF indicators were higher in the PP group for minimum meal frequency (44.8% vs. 37.9%), minimum dietary diversity (10.3 vs. 3.4%), iron-rich complementary foods (17.2 vs. 20.7%), and minimally acceptable diet (10.3% vs 3.6%), but differences were non-significant. Caregivers in the PP group demonstrated greater knowledge of healthful IYCF practices.

**Conclusions:**

Nutrition education can be effective to improve caregiver feeding practices and children’s dietary diversity and the frequency by which they are fed. A 10-week nutrition education and supplemental feeding program appears to provide some benefit to children in terms of dietary diversity and frequency of meals, and caregiver knowledge of feeding 1 to 2 months after program completion. However, children in this rural Ugandan region have diets that are still largely inadequate, highlighting the need for enhanced interventions and policies to promote diverse and appropriate diets for young children in this region. Future follow-up work in LNS-supported programs is recommended to understand how other similar approaches influence children's diet quality after program completion in other contexts.

## Background

Undernutrition is responsible for 45% of the 3 million annual preventable child deaths worldwide [1]. Poor nutrition in early childhood impairs cognitive development, learning and adult educational attainment and has only modestly decreased in Sub-Saharan Africa over the past 20 years [2, 3]. Reductions in undernutrition among infants and young children can be made through programmatic health and nutrition interventions [4]. Strategies such as nutritional counseling, food supplements, and cash transfers, delivered separately or in combination, aim to improve dietary outcomes and reduce undernutrition among food-insecure populations, with mixed results [5, 6]. Lipid-based nutrient (LNS) supplements can be an effective strategy for improving children’s dietary adequacy and growth and are sometimes delivered as part of holistic supplemental feeding programs that also deliver nutrition education along with other intervention such as growth monitoring, deworming, and education on sanitation and hygiene [7, 8]. These holistic programs provide short to medium-term food supplementation, seeking to correct or prevent nutrient deficiencies, with the ultimate of aim of preventing long-term undernutrition through improving dietary patterns.

The effect of LNS-supported nutrition education programs on caregiver feeding practices and children’s dietary adequacy after supplementation is discontinued is not well understood. In addition to the benefits gained from nutrition education, the potential of such programs to improve dietary adequacy relates to the possibility of an LNS product to change caregiver preferences for similar complementary foods (e.g. groundnut soup or thick porridges) that improve the nutrient density over children’s previous diets. Whether benefits from such programs are due to education, food supplementation, or a combination of both interventions, the long-term impact of complementary feeding interventions on children’s nutritional status depends largely on changes in the underlying household determinants of child feeding patterns, especially caregiver nutrition knowledge and the procurement of diverse diets for children [5].

Children in low-income countries are routinely fed diets that fail to meet global guidelines, especially in terms of the diversity of their diets and how often they are fed. A global analysis of countries with highest burden of undernutrition indicated that less than 40% of children in 16 of 22 sub-Saharan African countries in the survey were fed a “minimally diverse diet.” No country in sub-Saharan Africa had over 30% of children fed a diet that was “minimally acceptable.” Recent findings from Uganda indicate that lack of caregiver knowledge about healthy complementary feeding practices presents a major barrier to adequate child feeding [9]. Relatedly, in the most recent Ugandan national survey, caregivers with limited literacy skills were less likely to feed their children according to recommended infant and young child feeding (IYCF) practices [10].

Since 1995, World Harvest Mission (WHM, now called Serge) has been operating child nutrition programs in western Uganda to address the high prevalence of undernutrition. The goals of these programs have been to: 1) provide programs to prevent and treat malnutrition; 2) educate caregivers about healthy IYCF practices; 3) increase access to perinatal care resources; and 4) encourage food crop cultivation through demonstration gardens and seed distributions [11, 12]. Since 2008, WHM has operated the *Byokulia Bisemeye mu Bantu* (BBB) supplemental feeding program, which provides nutrition education, agricultural education, and a LNS supplement to underweight children (weight-for-age Z score < −2) ages 6 to 59 months.

To inform future programming and to understand the long-term influence of this program, we conducted a post-program follow survey to assess the impact of the BBB program on IYCF practices and caregiver’s knowledge of salient messages for IYCF.

## Methods

### Study design

We conducted a cross sectional study to compare dietary adequacy and caregiver feeding knowledge among caregiver-child dyads who participated in the BBB program and those that did not. We administered a household nutrition knowledge survey and 24-h dietary recalls among children who had completed the BBB program between January and July 2009, and a matched community comparison group.

### Setting

Bundibugyo is one of four districts in Uganda’s western region. At the time of the study, it was the only western district with no paved roads or electricity. The district is geographically isolated from Uganda due to its western border with the Democratic Republic of Congo, and eastern boundary with the Rwenzori mountains. The majority of families rely on subsistence farming, with a small export market for coffee and cocoa. Most cooking is done over open fire or with charcoal in metal grills.

The Bakonjo and Babwisi are the two predominant people groups in the 290,000-person district, which includes 52,500 (18%) children under 5 years. The prevalence of stunting (Height-for-age Z score (HAZ) < − 2) is 44%, compared to the national prevalence of 33% [13]. IYCF practices in Uganda present a major modifiable risk factor for reducing undernutrition and morbidity in children. Among children under 6 months, only 63% are exclusively breastfed. Among children 6 to 23 months, only 12.8% of children are fed 4 or more food groups per day, and just 5.8% are fed a minimally acceptable diet, which encompasses dietary diversity, feeding frequency, and being breastfed or fed milk products. Uganda woman face numerous challenges to their freedom to make decisions: nearly 40% of currently married women report that their husbands make the primary decisions about women’s health care and household purchases [13]. Constrained decision-making among women has been described to limit women’s caregiving capabilities for nutrition [14].

### Study tools

Based on previous qualitative interviews, we developed a quantitative survey and dietary recall instrument to assess caregivers’ nutrition knowledge and children’s diet quality [14]. Post-program (PP) caregivers were asked to recall key messages or topics that they received from the BBB program. Comparison Group (CG) caregivers were asked to recall any nutritional education messages they had ever received about child nutrition or feeding. Caregiver responses were coded in a table that listed pre-written IYCF messages, developed from UNICEF guidelines and the BBB curriculum [15]. Space was provided for answers outside of these pre-determined messages, and new categories were created to accommodate these responses.

Children’s diet adequacy was assessed through 24-h dietary recalls, and was obtained for each participating child immediately following the caregiver survey by study personnel trained in diet assessment methods. Two dietary recalls per child were collected on non-consecutive days in order to estimate the usual diet of children. The name and time of each meal and the ingredients and method of preparation for each food item were obtained in each recall. Next, the portion size offered and amount consumed by the child of interest were estimated using standard local utensils (e.g. tablespoon, 800 ml plate, 500 ml cup). Cups and plates were marked with fraction lines to assist caregivers in estimating portion sizes. Child ages were calculated by subtracting the birth date provided from a child health card, or from caregiver report when this was unavailable. Child weights were obtained through use of a hanging scale with a net for infants ≤12 months, or weighing pants for children >12 months.

### Assessment of infant and young child feeding (IYCF) practices

Among a subset of children ages 6 to 23 months, we used 24-h recall data to construct World Health Organization indicators for IYCF: continued breastfeeding at 12 months, feeding iron-rich complementary foods, minimum feeding frequency, minimum dietary diversity, and minimally acceptable diet [16]. Minimum meal frequency is defined as being fed solid or semi-solid foods the minimum number of times per day based on a child’s age. For breastfed children, this is twice for 6–8 months, three times for 9 to 23 months; non-breastfed children should be fed four or more times per day. Minimum dietary diversity is defined as being fed 4 or more food groups per day. A minimally acceptable diet is met for breastfed children if they were fed three or more food groups and were fed the age-specific minimum number of times per day (≥2 for children 6–8 months ≥ 3 for children ages 9–23 months). Non-breastfed children are required to consume a minimum of four food groups, consume milk or milk-based products, and be fed a minimum of four times per day. A food group was counted if a child consumed at least 1 g of a food item from any of the following seven groups: 1) infant formula, milk other than breast milk, cheese or yogurt, or other milk products; 2) foods made from grains, roots, and tubers, including matoke, porridge, fortified baby food from grains; 3) vitamin A-rich fruits and vegetables; 4) other fruits and vegetables; 5) eggs, meat, poultry, fish, shellfish and organ meats; 6) legumes and nuts; and 7) foods made with oil, fat, or butter [16]. Since we obtained two dietary recall observations per child, we indicated that an indicator was met if met on both days of recall. While this is a higher requirement than used in Demographic and Health Survey (DHS) assessments, we required that children meet the indicators on both days to assess usual diets more accurately.

### Study population

**Fig. 1:**
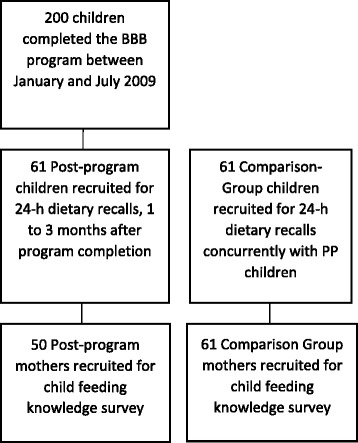
Study recruitment process

The study recruitment process is summarized in Fig. 1. PP caregivers were randomly selected from the list of program beneficiaries who were enrolled in the BBB program from different villages in the three sub-counties of Ndugutu, Bubandi, and Busaru between January and July 2009. We identified caregivers at their homes using program registries and recruited mothers to participate in a survey about their knowledge about child feeding and to measure their children’s diets on two days. All PP caregivers who participated in the 10-week BBB Program between January to July 2009 were eligible for recruitment. All study participants were recruited for the post-program study between August and October 2009. All PP caregivers were recruited four to eight weeks after program participation, one to two months after LNS supplements and education were discontinued. Per normal protocol, all caregiver contact with the program was discontinued after 10 weeks of participation; children who fail to make improvements in growth were referred to the health center for examination by physician and potentially in-patient treatment. Research teams contacted the local chairperson in each village to explain the study purpose, share the human subject’s approval, and to obtain additional approval from these local leaders to speak with caregivers in their jurisdiction. All surveys and dietary recalls were conducted in either Lubwisi or Lukonjo, the two primary local languages, depending on the caregiver preference. All caregivers of children in the CG, and 50 caregivers of PP children completed the child feeding survey, for a total of 111 survey respondents. Eleven of the 61 PP participants were unavailable for the child feeding survey. There were no identifiable differences between respondents and non-respondents for the child feeding survey.

### Comparison group

We recruited a post-test only comparison group (CG) from the same source population (villages) that produced the PP group to compare dietary adequacy and caregiver feeding knowledge [17]. Since PP children had recently participated in a 10-week program designed for children who were underweight, and which sought to enroll most of the underweight children in their communities, we elected to instead match children based on age and village of residence. We obtained a list of households with children of similar ages (±2 months) to the PP children from the local chairpersons in each village. CG caregivers were then randomly sampled from this list with one-week lag to the PP group. This method enabled the PP and CG groups to be similar in age and broader socio-economic characteristics that are largely homogenous within villages. This study design assumes that secular trends in infant feeding knowledge or practice would have been trivial in one to three months of time, and that there was minimal spillover from program messages from the program group to the broader community.

All interviews were conducted in participant homes and lasted approximately 30 min. No eligible participants who could be located refused participation. Per study protocol, the informed consent process was administered orally and with a two-page form. Written consent was obtained from subjects who could write their names. Mothers who could not write their names wrote an “X” to indicate written consent.

### Program components of the BBB supplemental feeding program

The BBB program operated in two health centers in the Bundibugyo District and enrolls 50 children and their caregivers per 10-week cycle. The program delivered complementary feeding education to caregivers and 650 kcal/day (128 g/day) of a peanut and soy-based LNS to supplement the diets of underweight children (WAZ < −2). For children ages 12–36 months, the daily ration provided the full estimated average requirement (EAR) for vitamins A, folate, and zinc, and approximately 50% for calcium and vitamin C [11]. Previous evaluation of a different cohort of program participants indicated that the food supplements were well-received and incorporated into the normal child feeding routine [18].

At each weekly visit caregivers received child growth monitoring, nutrition education, and the LNS. Village health workers and health center staff delivered the education, which was based on UNICEF IYCF guidance. The program emphasized 1) the impact of early nutrition on school performance later in life, 2) antenatal nutrition, 3) growth monitoring and promotion, 4) exclusive breastfeeding through 6 months and continued breastfeeding on demand after 6 months; 5) introduction of complementary foods at 6 months; 6) feeding a diverse diet that includes animal source foods; complementary feeding practices, 7) using an attentive, responsive child feeding style, 8) feeding children during and after illness, and 9) appropriate hygiene and sanitation practices [12, 15].

### Hypothesis and statistical analysis

IYCF indicators, practices, and caregiver knowledge of child feeding practices were compared using two-sample proportion and mean comparison tests (two-tailed) using STATA version 14.0 [19]. We hypothesized that a greater proportion of PP children would meet the recommended IYCF indicators, and that the proportion of mothers who recalled specific healthful child feeding practices would be greater in the PP group compared to the CG group. Group proportion differences controlled for underweight status, presence of the father in the home, maternal education, primary means of food acquisition, type of material used to construct the respondent’s home. These confounders were selected because they were potentially associated with the exposure (group membership), independent risk factors for the outcome (IYCF practices and knowledge), do not lie in the causal path between the exposure and the outcome, and could not be a result of the outcome. The sample size was calculated based on the assumptions of α = 0.05, β = 80%, and a two-tailed test in order to detect a 20% difference in the proportion of children who were fed a minimally acceptable diet. Based on these parameters, a total sample of 118 was required, with 59 children per study group.

### Ethical approval

This study was conducted according to the guidelines established in the Declaration of Helsinki. Per study protocol, the informed consent process was administered orally and with a two-page form. Written consent was obtained from subjects who could write their names. Mothers who could not write their names wrote an “X” to indicate written consent. All procedures involving human subjects are approved by the University of North Carolina School of Public Health Institutional Review Board, # 08–1100 and the Bundibugyo District Health Office, which provided local ethical approval of the study.

## Results

A total of 122 children were recruited for the study, with 61 children in each study group. Demographic characteristics (Table 1) and dietary recall information were obtained for all 122 study participants.

**Table 1:** Demographic information of study participants, according to study group^a^

	Post-Program *N* = 61	Comparison *N* = 61	*P*-value (2-tail)
Child age in months (mean ± SE)	26.8 ± 1.6	23.4 ± 1.9	0.184
Percent underweight, Weight-for-age Z-score < −2 ^b^	44 (72%)	24 (39%)	< 0.001^c^
Percent male	34 (56%)	33 (55%)	0.856
Percent with living father	58 (95%)	54 (86%)	0.088
Percent with father present in the home	26 (42%)	49 (80%)	< 0.001^c^
Highest Paternal Education (mean ± SE)	6.9 (0.4)	6.0 (0.6)	0.236
None or some primary	38 (62%)	37 (61%)	0.852
Some secondary or higher	23(38%)	24 (39%)	0.464
Percent with living mother	59 (97%)	59 (97%)	1.000
Percent with mother or grandmother present in the home	58 (95%)	55 (90%)	0.299
Highest Maternal Education (mean years ± SE)	3.0 ± 0.4	3.6 ± 0.4	0.290
None	23 (38%)	24 (39%)	0.852
Some primary	34 (56%)	32 (52%)	0.716
Some secondary or higher	4 (6%)	5 (9%)	1.000
Building Materials			
Percent with tin roof	9 (15%)	10 (16%)	0.803
Percent with mud walls, no cement or bricks	54 (89%)	55 (90%)	0.769
Number of birth children (mean ± SE)	4.2 ± 0.3	3.8 ± 0.3	0.257
Caregiver marital status			
Married, monogamous	25 (41%)	45 (73.8%)	0.003^c^
Married, polygamous	29 (48%)	12 (20%)	0.001^c^
Separated, divorced, or widowed	7 (11%)	4 (6.6%)	0.093

^a^Study sample includes *n*= 61 maternal-child dyads in the Post-Program and *n*=61 maternal-child dyads in the Community Comparison group, *N*=122

^b^﻿Weight-for-age Z-score calculated according to the 2006 World Health Organization Multicenter Growth Reference Study

^c^Result was significant, *﻿p﻿* < 0.05

The PP children were more likely to be underweight (72 vs. 39%, *p <* 0.001), and to live in households with no father present (58 vs. 20%, *p <* 0.001). Mothers of PP children were also more likely to be co-wives in polygynous unions (48 vs 20%%, *p <* 0.001). PP children were less likely to have a father present full-time in their homes (42 vs. 80%, *p <* 0.001). Parental education and fertility rates were similar between groups. Overall, the combined study sample was very disadvantaged: 89.5% lived in homes with mud walls, and only 15.5% had tin roofs. The mean maternal education was 3.3 years.

### Comparison of infant and young child feeding (IYCF) practices

Dietary diversity was calculated for the full (*n* = 122) sample, while IYCF indicators apply only to children 6 to 24 months (Table 2). Therefore, these indicators were constructed on a subset of children (*n* = 58), based on two observations per child. A mean (±SE) comparison test indicated that children in the PP group had a significantly greater Dietary Diversity Score (DDS) compared to CG children (3.0 ± 0.1 vs. 2.0 ± 0.2, *p =* 0.002). This difference remained significant after controlling for underweight status, presence of the father in the home, maternal education, primary means of food acquisition, type of material used to construct the respondent’s home (*p =* 0.001). Caregivers in the PP group reported a greater number of mean feeding occasions than caregivers in the CG (2.8 ± 0.2 versus 2.2 ± 0.2, *p =* 0.004) which remained significant after controlling the same confounders (*p =* 0.003). PP children were more likely to be breastfeeding at 12 months (72.3 vs. 16.7%, *p <* 0.027); however, this result was no longer significant after adjustment.

**Table 2:** Comparison of Infant and Young Child Feeing Indicators and practices between Post-Program and Comparison groups^a^

Feeding Practice	Post-Program	Comparison	P-value (2-tail)	P-value Adjusted^b^
Mean number of feeding occasions ± SE	3.0 ± 0.1	2.1 ± 0.2^c^	0.002^﻿c^﻿	0.001^c^
Mean dietary diversity ± SE	2.8 ± 0.2	2.2 ± 0.2	0.004^c^	0.003^c^
Continued breastfeeding at 12 months	72.3%	16.7%	0.027^c^	0.166
Fed minimum meal frequency	44.8%	37.9%	0.594	0.612
Fed minimum dietary diversity	10.3%	3.4%	0.300	0.424
Fed iron-rich complementary foods	17.2%	20.7%	0.737	0.336
Fed a minimally acceptable diet	10.3%	3.4%	0.300	0.262

^a^Infant and Young Child Feeding Indicators are calculated for all children ages 6 to 24 months (n = 58, of which 29 are in each study group) based on two 24-h recall observations per child (n = 116 observations total)

^b^Adjusted for presence of father in the home, maternal education, primary means of food acquisition, type of material in respondent’s home, and underweight status of the child

^c^Result was significant, *p* < 0.05

Children in the PP group were more likely to be fed the minimum meal frequency (48.5% vs 37.9%), minimum dietary diversity (10.3 vs 3.4%), iron-rich complementary foods (17.2 vs. 20.7%), and a minimally acceptable diet (10.3 vs. 3.4%). These differences were non-significant after adjustment for potential confounders (Table 2).

Compared to CG children, PP children were more likely to consume foods from all of the food groups assessed; however only “Other fruits and vegetables” (95.1 vs. 53.3%, *p =* 0.000), “meats, poultry, and fish” (39.3% vs. 31.7%, *p =* 0.010), and “fats/oils” (88.5 vs. 63.3%, *p =* 0.001) were significantly different (Table 3). Of the non-breastfed children, no participants in either group were fed milk products.

**Table 3:** Comparison of food groups fed to Post-Program and Comparison group children in the previous 24-h based on two days of recall^a^

	Post-Program (*N* = 61)	Comparison (*N* = 61)	P-value (2-tail)	P-value Adjusted^b^
Cereals, roots, tubers, and matoke	98.3%	91.7%	0.090	0.126
Vitamin A-Rich fruits and vegetables	8.2%	5%	0.479	0.747
Other vegetables	95.1%	53.3%	< 0.001^c^	<0.001^c^
Legumes, pulses, nuts	65.6%	50%	0.083	0.836
Meat, poultry, fish	39.3%	31.7%	0.377	0.010^c^
Fats, oils	88.5%	63.3%	0.001^c^	0.013^c^
Dairy	1.6%	3.2%	0.559	0.079
Eggs	3.3%	0%	0.157	NA^d^

^a^Value shown are number (percentage). Individuals were considered to consume a food group if a minimum of 1 g was consumed on the first day of recall

^b^Adjusted for presence of father in the home, maternal education, primary means of food acquisition, type of material in respondent’s home, and underweight status of the child

^c^Difference was significant, *p* < 0.05 for a two-tailed test of proportions

^d^NA Adjust not possible since group member predicts outcome perfectly for egg consumption

### Comparison of caregiver recognition of infant and young child feeding information

Survey results indicated that PP caregivers had greater knowledge of key behaviors emphasized in the program after the program was discontinued compared with the control group of children (CG) (Table 4). The most commonly cited messages are identified in ranked order. Specific messages that were significantly different between groups and higher in the PP group were “How often to feed my child,” “Feeding different kinds of foods,” “Feeding soft foods” “The importance of breastfeeding,” “Growing more foods in my garden that my child can eat,” and “Feeding more sauce than food.”

**Table 4:** Comparison of recall of nutrition education topics and messages by post-program caregivers and CG groups^1^

	PP *n* = 50	CG *n* = 61	*P*-value (2-tail)	*P*-value adjusted^b^
Washing children’s hands before feeding, general hygiene messages	41 (82%)	0 (0%)	<0.001^c^	NA
Feeding different kinds of foods	35 (70%)	12 (20%)	<0.001^c^	<0.001^c^
Not adding too much water to the child’s meals	27 (54%)	0 (0%)	<0.001^c^	NA
How often to feed infants and young children	27 (54%)	10 (16%)	<0.001^c^	0.001^c^
Feeding a small amount of food that my child can finish	23 (46%)	0 (0%)	<0.001^c^	NA
The importance of breastfeeding	18 (36%)	7 (11%)	0.002^c^	0.009^c^
Feeding more “sauce” than food to provide adequate protein	14 (28%)	1 (2%)	<0.001^c^	0.010^c^
Growing foods in my garden that my child can eat	13 (26%)	1 (16%)	<0.001^c^	0.005^c^
Feeding soft foods	11 (22%)	9 (15%)	0.323	0.480
Monitoring/being attentive to my child when she eats	8 (16%)	0 (0%)	<0.001^3^	NA
Feeding during and after my child gets sick	5 (10%)	7 (11%)	0.803	0.199

^a^Values shown are number (proportion as a percentage of group total)

^b^Adjusted for presence of father in the home, maternal education, primary means of food acquisition, type of wall construction material in respondent’s home, and underweight status of the child

^c^Result was significant, *p* < 0.05

## Discussion

Caregivers in Uganda who participated in a 10-week supplemental feeding and nutrition education program fed their children higher quality diets, and reported more comprehensive knowledge of healthful IYCF practices than those with no such program exposure. Despite this comparative success, the majority of children in both study groups were still not fed according to IYCF guidelines. Education interventions that improve linear growth, which was not assessed in the present study, have typically directed caregivers to feed diverse complementary foods and have encouraged the consumption of animal source foods [20–22]. Notably, less than 40% of children in the PP group consumed animal source foods, and just over 30% in the CG group.

While children in the PP group achieved, on the whole, more adequate diets compared to CG children, a higher proportion of PP children were classified as underweight. This difference most likely reflects the original enrollment criteria of the BBB program, which recruited children based on their underweight status. We have previously documented challenges to feeding children adequately during supplementation in this program [11]. Moreover, there is a considerable biologic challenge to reversing the effects of malnutrition after 24 months, although attenuation of growth deficits is possible after in conditions where economic circumstances, diet, and health improve [23]. The fact that our study sample had a much higher prevalence of underweight than regional estimates of the 2011 DHS likely reflects a more nutritionally disadvantaged population in the Bundibugyo region compared to entire western region, and that the PP group was previously enrolled in a program for underweight children.

The feeding practices of children in the present study were comparable, or slightly poorer, to DHS estimates in the study region. For example, 30.8% of children in 2011 DHS consumed iron-rich foods, compared to 30.7% in the PP group and 24% in the CG group in the present study. The 2011 DHS western region estimate for dietary diversity is 15.3%, and 55.5% for minimum meal frequency. The percent of PP children in the present study who met the minimally acceptable diet indicator was 17.2%, markedly higher than both the DHS (5.9%) and CG estimates (6.9%), although still much lower than in comparable settings with moderate food insecurity and high undernutrition such as Ecuador, Ethiopia, Malawi, and Vietnam [24–27]. Feeding practices in this region of Uganda have major room for improvement, and will require multi-sectoral efforts from a variety of organizations from the health, agricultural, education and child development sectors.

To our knowledge, no published studies report feeding practices of children following participation in a LNS-supported program. This study demonstrates that even after short-term supplementation of LNS is discontinued, some improvements in IYCF practices are sustained. Our study provides evidence of improved feeding practices and caregiver IYCF knowledge from an on-going community program that is operated by a small-scale NGO and community members. Several recent studies report a sustained impact from educational programs that were similar to the one assessed in this study. A complementary feeding education trial in Malawi that delivered four nutrition education lessons reported improved adoption of learned practices, greater dietary intake, and improved quality of complementary foods among intervention recipients ages 6 to 23 months [28]. A cluster randomized trial in Laishui, China that delivered complementary feeding education through group training or home visits for 2–4 months found that mothers assigned to the intervention group exhibited substantial changes in complementary feeding, and that children exhibited improved growth over controls after one year [20]. A community health worker-delivered intervention in Haryana, India provided complementary feeding and nutrition counseling to mothers of newborns and found that energy intake and length gain were significantly improved, while weight gain was not, compared to controls [28]. In a rural Ethiopia region with a high stunting prevalence, mothers who had access to nutrition education had better knowledge of IYCF practices and lower rates of child stunting [29]. Thus, education can be an effective method for improving IYCF, even in regions of high food insecurity and growth faltering.

The recall of nutrition education topics by these two groups of caregivers provided insight into how this 10-week program influenced caregiver internalization of key child feeding topics, and also highlighted areas where mothers who were not exposed to a supplemental feeding and nutrition education program were particularly uninformed. Topics such as hygiene, avoiding excessive dilution of children’s meals, feeding small quantities of food that children can finish, and being attentive to children during feeding were mentioned by few or no mothers in the CG. While our study did not assess in what these messages meant to caregivers, or how they were put into practice, it was noteworthy to observe that four of 10 main IYCF messages were reported by a majority of caregivers. Based on discussion with program delivery staff, the messages that were most commonly reported by PP caregivers in this study were messages reinforced with visual imagery, such as food preparation or child care demonstrations that accompanied verbal instruction. Visual aids and demonstrations have been reported by health education workers as an effective method of conveying IYCF information [30].

The relatively high frequency that PP caregivers noted to feed more “sauce than food” and to avoid dilution of children’s meals with excessive water suggest that, beyond the direct nutritional benefits of LNS, the energy-dense product may be a suitable example complementary food that can be used concurrently with education. Beyond its usefulness as a demonstration complementary food, LNS remains an important product to address gaps in micronutrient quality of complementary foods, as does supplementation or fortification of local complementary foods [31]. While education appeared to have lasting benefits to children’s diets, most studies conclude that education only versus education combined with food supplements are more effective and reducing undernutrition [32–34].

Our study was limited by several factors. First, despite efforts to compose a comparison group that was representative of the source population of post-program participants, our groups differed in two ways: marriage patterns and underweight status. These differences suggest that the PP group may have been less food secure as another study in the same region found that female caregivers rely on children’s’ fathers for financial support to purchase food, and mothers who are co-wives are more nutritionally vulnerable [14]. Thus, the improved dietary practices documented in the PP group should be interpreted in the context of potentially lower food security in this group. Second, since this study did not apply a factorial design to examine education and LNS as separate factors for promoting dietary change, it is not possible to determine the distinct roles of the LNS and nutrition education on feeding practices. It is plausible that the local LNS used in the nutrition program served a model complementary food that, when delivered with nutrition education, facilitated improved dietary patterns and better dietary quality. Third, our study did not collect height measurements. This was due to budget constraints at the time of the study. This omission eliminated the option to compare HAZ and WLZ which would provide additional insight into between group anthropometric differences. We focus the comparisons on child feeding practices since this study was primarily focused on dietary practices after program completion. Fourthly, we contacted mothers four to eight weeks after program participation ended, and therefore the recall of program information and the corresponding impact on feeding practices across this time period may vary based on the different amount of time that lapsed since contact with the program ended. Finally, the use of a post-program only comparison group is less scientifically rigorous than a true randomized comparison, or to a study design that collected pre-intervention measures. While these approaches would be more rigorous, the study design used in the present study was selected to provide timely feedback to an ongoing program to understand how caregivers were feeding following program participation compared to peers in the community.

Because the PP group was potentially less food secure based on the differences in underweight status, we expect the direction of bias to demonstrate poorer feeding practices among the PP group. While the potential exists for social desirability bias, we expect this bias to be similar between groups since the evaluation team was not involved in the program delivery, and since program participation was completed. We ensured all participants that they study purpose was to understand how children in Bundibugyo are fed, and what their mothers know about feeding young children, with the goal of improving child nutrition programs in the district.

The study sample size was calculated to detect a 20% difference in the probability of children being fed a minimally acceptable diet; however, the actual difference in these estimated was only 11.3%. There, the statistical significance of the findings for IYCF practices is best interpreted as trends to demonstrate improved feeding practices among the PP group. Future studies to extend these research findings will apply more rigorous research designs that include larger sample sizes.

## Conclusions

Participation in a combined nutrition education and supplemental feeding program in rural Uganda confers some lasting benefits to IYCF practices and the corresponding dietary adequacy of children, one to 3 months after free supplemental food rations are discontinued. The potential long-term impacts of supplemental nutrition interventions can be strengthened through program components that address child-feeding practices using low-cost, community-based approaches. Future studies should examine the separate and combined effects of LNS and nutrition education on feeding practices and growth outcomes, and should evaluate varying intensities of IYCF education to inform community-based prevention efforts in a variety of contexts.

## Abbreviations

BBB: Byokulia Bisemeye mu Bantu
CG: Comparison Group
DHS: Demographic and Health Surveys
HAZ: Height-for-Age Z score
IYCF: Infant and Young Child Feeding
LNS: Lipid-based Nutrient Supplement
mo: Months
PP: Post Program
UNICEF: United Nation’s Children’s Fund
WAZ: Weight-for-age Z score
WHM: World Harvest Mission
WHO: World Health Organization
